# A GIS-based extended fuzzy multi-criteria evaluation for landslide susceptibility mapping

**DOI:** 10.1016/j.cageo.2014.08.001

**Published:** 2014-12

**Authors:** Bakhtiar Feizizadeh, Majid Shadman Roodposhti, Piotr Jankowski, Thomas Blaschke

**Affiliations:** a Department of Remote Sensing and GIS, University of Tabriz, Tabriz, Iran; b Department of GIS and Cartography, University of Tehran, Tehran, Iran; c Department of Geography, San Diego State University, San Diego, CA, United States; d Institute of Geoecology and Geoinformation, Adam Mickiewicz University, Poznan, Poland; e Department of Geoinformatics—Z_GIS, University of Salzburg, Salzburg, Austria

**Keywords:** GIS based MCDA, Fuzzy-AHP, Membership functions, Landslide susceptibility maps, Izeh River basin

## Abstract

Landslide susceptibility mapping (LSM) is making increasing use of GIS-based spatial analysis in combination with multi-criteria evaluation (MCE) methods. We have developed a new multi-criteria decision analysis (MCDA) method for LSM and applied it to the Izeh River basin in south-western Iran. Our method is based on fuzzy membership functions (FMFs) derived from GIS analysis. It makes use of nine causal landslide factors identified by local landslide experts. Fuzzy set theory was first integrated with an analytical hierarchy process (AHP) in order to use pairwise comparisons to compare LSM criteria for ranking purposes. FMFs were then applied in order to determine the criteria weights to be used in the development of a landslide susceptibility map. Finally, a landslide inventory database was used to validate the LSM map by comparing it with known landslides within the study area. Results indicated that the integration of fuzzy set theory with AHP produced significantly improved accuracies and a high level of reliability in the resulting landslide susceptibility map. Approximately 53% of known landslides within our study area fell within zones classified as having “very high susceptibility”, with the further 31% falling into zones classified as having “high susceptibility”.

## Highlights

• We have developed a new multi-criteria decision analysis method for landslide mapping.
• Our method is based on fuzzy membership functions derived from GIS analysis.
• We integrated extended fuzzy with AHP to overcome of associated uncertainty with GIS-Multicriteria Decision Making.
• The results demonstrate that the accuracy of GIS-Multicriteria can be improved through FAHP.

## 1 Introduction

Landslides are destructive natural hazards that frequently lead to loss of human life and property, as well as causing severe damage to natural resources (Intarawichian and Dasananda, 2010, Feizizadeh and Blaschke, 2013a). Landslide susceptibility mapping (LSM) is considered to be an effective tool for understanding these natural hazards and predicting potential landslide hazard areas (Feizizadeh and Blaschke, 2013a), thereby mitigating their impacts. LSM addresses how likely a terrain is to produce slope failures, with susceptibilities expressed cartographically in maps that portray the spatial distribution of future slope-failure susceptibility (Lei and Jing-feng, 2006, Feizizadeh and Blaschke, 2013a, Feizizadeh et al., 2013a).

LSM requires a multi-criteria approach and high levels of accuracy and reliability in the resulting maps, in order to be relevant for decision making and the design of disaster management plans. The effectiveness of decision making is clearly dependent on the quality of the data used to produce the landslide susceptibility maps, as well as on the method used for decision-making analysis. GIS-based multicriteria decision analysis (MCDA) is considered to be an important spatial analysis method in the decision-making process that allows information derived from different sources to be combined (Feizizadeh and Blaschke, 2001). GIS-based MCDA is an intelligent approach to converting spatial and non-spatial data into information that can, together with the decision maker’s own judgement, be used to assist in making critical decisions (Chen et al., 2010, Sumathi et al.,, Gbanie et al., 2013). GIS based MCDA provides a collection of powerful techniques and procedures for dealing with decision-making problems and for designing, evaluating, and prioritizing possible alternative courses of action (Feizizadeh and Blaschke, 2013a, Feizizadeh and Blaschke, 2012, Feizizadeh et al., 2012). GIS integrated with MCDA methods provide a framework within which to handle different aspects of the various elements of a complex decision-making problem, to organize the various elements into a hierarchical structure, and to study the relationships between these different components of the problem (Malczewski, 2006).

Methods of MCDA can be subdivided into Multiple Attribute Decision Making (MADM) and Multiple Objective Decision Making (MODM) (Malczewski, 1999a). Even though the distinction is derived from two specific meanings: attribute and objective, of a generic term: criterion (pl. criteria) the dichotomy of MCDM goes beyond the semantics of criterion. The MADM approach requires that the choice (selection) be made among decision alternatives described by their attributes, where criteria are derived from attributes. MADM problems are assumed to have a predetermined, limited number (tens or hundreds) of decision alternatives. Accordingly, in this paper we focus on multiple criteria evaluation of land units and their susceptibility to landslides. Multiple criteria evaluation involves a set of quantifiable spatial criteria, their standardization functions, techniques for expressing preferences regarding the relative importance of the criteria, and aggregation rules combining quantified criterion preferences with standardized criterion values into an overall evaluation score. This procedure makes multiple criteria evaluation especially attractive for integration with GIS for the purpose of solving spatially-explicit land allocation/land use problems (Carver, 1991, Jankowski, 1995, Malczewski, 2004, Chakhar and Mousseau, 2008, Chen and Paydar, 2012).

An analytic hierarchy process (AHP) is one of the GIS-MCDA methods which have been successfully applied to many decision maker systems (Lai, 1995). In spite of AHP’s popularity, the method is sometimes criticized for its inability to adequately handle the inherent uncertainties and imprecisions associated with the mapping of a decision-maker’s perception to crisp numbers (Chen et al., 2011). The AHP׳s pairwise matrix is based on expert opinion and thus introduces a degree of subjectivity when used to make comparison judgments. Any incorrect perception of the roles of the different criteria on behalf of the expert can consequently easily be conveyed into the assignment of weightings (Kritikos and Davies, 2011, Feizizadeh and Blaschke, 2013b). AHP can be integrated with fuzzy logic methods in order to deal with this source of uncertainty and to provide a framework for further analysis that makes use of the advantages of fuzzy membership functions (FMFs) to assess criteria and improve the accuracy of the results.

Fuzzy sets have been applied in the context of MCDA in order to standardize criterion maps by assigning to each object a degree of membership or non- membership of each of the criteria (Jiang and Eastman, 2000, Gorsevski and Jankowski, 2010). Combining an AHP with fuzzy set theory permits greater flexibility in the assessment of results and the subsequent decision making. A fuzzy-AHP (FAHP) retains many of the advantages enjoyed by conventional AHPs, in particular the relative ease with which it handles multiple criteria and combinations of qualitative and quantitative data. As with an AHP, it provides a hierarchical structure, facilitates decomposition and pairwise comparison, reduces inconsistency, and generates priority vectors. Finally, an FAHP is able to reflect human thought in that it uses approximate information and uncertainty to generate decisions (Kahraman et al., 2004). These characteristics qualify the use of an FAHP as an appropriate and efficient tool to assist with making complex decisions in environmental management (Vahidnia et al., 2009).

Fuzzy set theory employs a membership function that expresses the degree of membership value with respect to a particular attribute of interest. The attribute of interest is generally measured over discrete intervals and the membership function can be expressed as a table relating map classifications to membership values (Pradhan, 2010, Pradhan, 2011a, Pradhan, 2011b). Fuzzy logic is straightforward to understand and to implement, and has been successfully integrated with GIS-MCDA. GIS-based MCDA can be used together with fuzzy set theory to model imprecise objectives in a variety of research areas (Chang et al., 2008, Yonca Aydin et al., 2013), especially for landslide susceptibility mapping purposes (Akgun et al., 2012, Shadman et al., 2013). Technically, the fuzzy logic method leads to a flexible combination of weighted criteria that can subsequently be implemented through GIS-MCDA, in order to further improve the accuracy of results (Pradhan, 2010, Pourghasemi et al., 2012). GIS-MCDA technic differs from data-driven approaches, such as weights-of-evidence methods or logistic regression, in that it uses the locations of known objects such as landslides to estimate weightings or coefficients (Pradhan, 2011a, Pradhan, 2011b, Pourghasemi et al., 2012). Since the LSM process deals with a variety of criteria it can be assumed that integration of fuzzy set theory with MCDA, and in particular with an FAHP, will lead to improvements in the accuracy of landslide susceptibility maps due to the flexibility of fuzzy membership functions. Based on this assumption, the main objective of this research was to develop a new approach for tackling uncertainty and imprecision within the analytical hierarchy prioritization process by representing the decision-maker’s judgments as fuzzy numbers or fuzzy sets. In order to achieve this objective we used an FAHP to develop a landslide susceptibility map of the Izeh Basin in Iran, which is highly prone to landslide hazards.

## 2 Study area

**Fig. 1:**
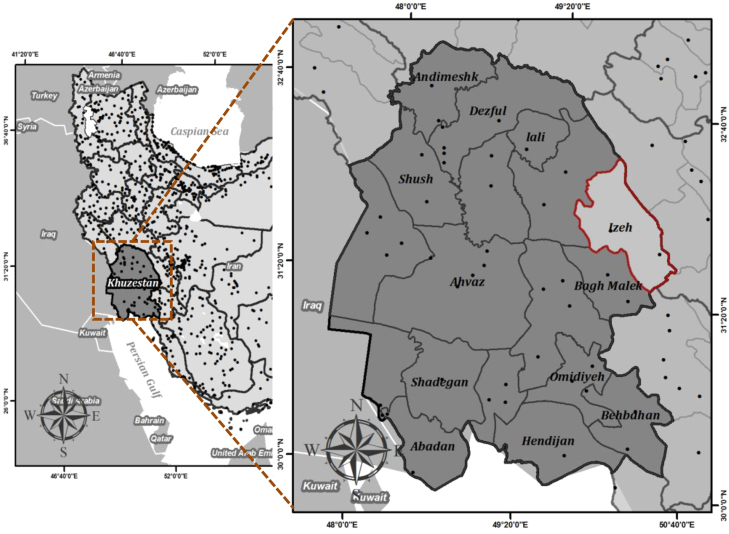
Location of the study area.

The study area lies within the Izeh Basin, which covers an area of 3929.78 km^2^ in the Khuzestan province of south-western Iran (see Fig. 1). The elevation in the Izeh Basin ranges between 342 m and 3579 m above sea level. The area has a temperature climate and the annual precipitation ranges from 400 mm in the lowest areas to 800 mm in the mountains. The Izeh Basin is important in terms of its agricultural production and, in particular, its hydroelectric power plants. The Karun River, which is the longest river in Iran, flows through this basin. The canyons that the Karun River flows through have provided opportunities for the construction of hydroelectric power plants and three main dams have been built to date along different stretches of the Karun River. The area is, however, highly susceptibility to mass movements, and in particular to landslides, which are considered to represent a potential hazard to the hydropower plants of the Izeh Basin.

The geology of the area is very complex and landslides are mostly common within Quaternary pediment fan, Asmari and Aghajari formations. The landslide inventory database for Izeh Basin records 106 landslide events which had occurred prior to 2013 and recorded posterior this date. Tectonic activity, combined with the presence of sedimentary formations such as marls, shales, limestones, gypsum, and siltstones, render this area highly susceptible to landslide hazards.

## 3 Material and methods

We used the following three step methodology for LSM: (a) collection of data and establishment of a spatial database, from which the causal landslide factors are then extracted, (b) assessment of landslide susceptibilities using the relationships between landslides and their conditioning factors, and (c) validation of results. The methodology that we used consisted of two stages. The first stage involved the integration of an AHP with fuzzy set theory in order to make use of the advantages of fuzzy set theory in AHP-based pairwise comparisons for qualitative analysis and reducing the subjectiveness inherent in the assessment of criteria weights. Expert opinion was sought to rank the criteria on the basis of their importance and the criteria weights were then calculated using fuzzy pairwise comparisons. The second stage in our methodology involved the application of the FMFs to results from the first stage in order to calculate the fuzzy membership values for each landslide conditioning factors.

### 3.1 Selection of criteria and data processing

**Fig. 2:**
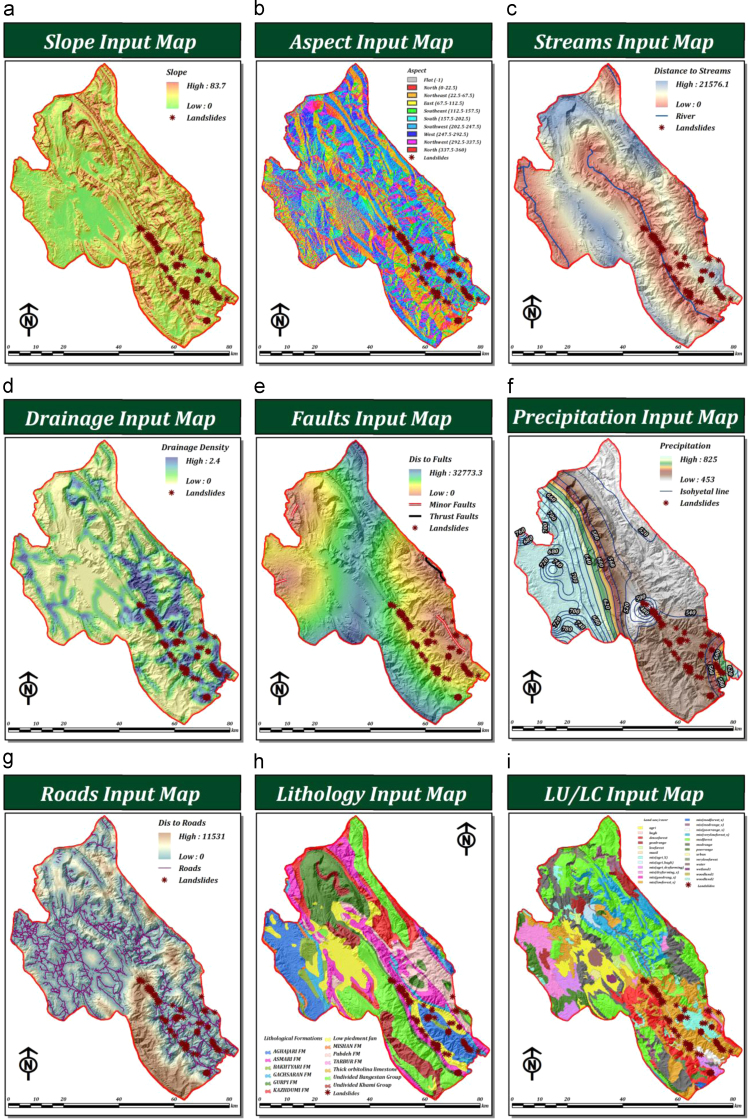
Spatial distribution of the selected criteria: (a) slope, (b) aspect, (c) distance to streams, (d) drainage density, (e) distance to faults, (f) precipitation, (g) distance to roads, (h) lithology, and (i) land use/land cover.

In order to generate a landslide susceptibility map, criteria need to be identified that are relevant to the particular situation under consideration. The set of criteria selected should adequately represent the problem domain and should contribute towards the ultimate objective (Prakash, 2003, Feizizadeh and Blaschke, 2013b). For our research we selected four main criteria (topography, hydrology, climate, and human factors) and nine sub-criteria (slope, aspect, distance to streams, distance to roads, drainage density, distance to faults, lithology, precipitation, and land use/land cover). Fig. 2 shows the spatial distributions of the selected sub-criteria.

A number of different data sets were used to prepare the selected criteria and for input into the evaluation model. The lithology and fault data were derived from published 1:100,000 geological maps. The road and streams data were extracted from 1:50,000 topographic maps of the study area, which were used to create digital elevation models (DEMs) that were in turn used to derive the slope and aspect data. The land use/land cover data was derived from Landsat ETM+ satellite imagery with a 30 m spatial resolution through image processing techniques. Available meteorological data were used to derive annual average precipitation figures for the precipitation map, using interpolation methods in GIS. The landslide inventory database of the study area, which was recorded by field survey using GPS locations (MNR, 2010), was used for validation of the final landslide susceptibility map. All of the data preprocessing and standardization of the selected LSM criteria required in the preparation phase was performed on the original datasets in Arc GIS software, prior to further analysis and implementation of fuzzy-MCDA.

### 3.2 Fuzzy set theory

Fuzzy set theory is widely used as a modeling approach for complex systems that are difficult to define exactly in crisp numbers. The theory was introduced by Zadeh in 1965. Fuzzy logic permits the input of vague, imprecise, and ambiguous information (Kahraman and Kaya, 2010, Balezentiene et al., 2013). Fuzzy logic is commonly used in spatial planning in order to be able to treat the spatial objects on a map as members of a set. In a classic case which is sometimes called “crisp” an object either belongs to a set or not. However, in fuzzy set theory a candidate objects can take on membership values between 0 and 1 which reflects a degree of membership (Zadeh, 1965).

**Fig. 3:**
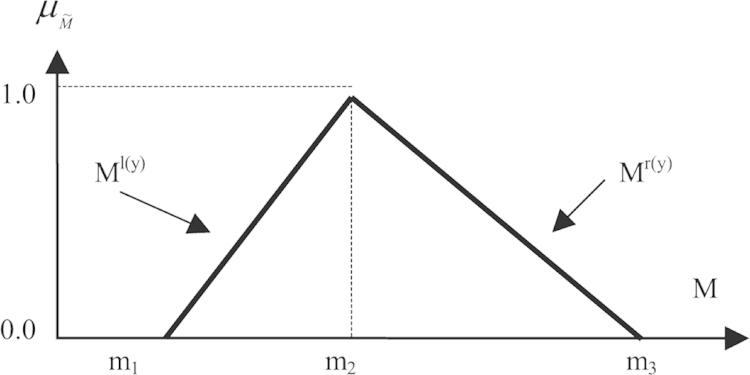
A fuzzy triangular number (Kahraman et al., 2003).

A fuzzy set can be described as follows: if *Z* denotes a space of objects, then the fuzzy set (*A*) in (*Z*) is a set of ordered pairs:

(1) A{z,MF(z)},z∈Z

where the membership function MF(z) is the set *A*’s degree of membership to *Z*. Fig. 3 shows the triangular fuzzy number (TFN)$\;\tilde{M}$ contains the basis for the membership function the TFNs are denoted simply by *m*_1_, *m*_2_, and *m*_3_. The parameters *m*_1_, *m*_2_ and *m*_3_ respectively denote the smallest possible value, the most promising value, and the largest possible value that describes a fuzzy object (Kahraman et al., 2003). Using this approach each TFNs has a linear representation on its left and right sides and the membership function can be defined as:

(2) $$\mu \;(x|\tilde{M})\{\begin{array}{ll} 0, & x<m1\\ (x-m_{1})/(m_{2}-m_{1}), & m_{1}\leq x\leq m_{2}\\ (m_{3}-x)/\;(m_{3}-m_{2}), & \;m_{2}\leq x\leq m_{3}\\ 0, & x<m_{3} \end{array}$$

A fuzzy number can always be assigned based on its corresponding left and right representation of each degree of membership (Kahraman et al., 2003):

(3) $$\tilde{M}=(M^{l(y)},M^{r(y)})=(m_{1}+\;(m_{2}-m_{1})y,\;m_{3}+(m_{2}-m_{3})y).y\in [0,1]$$

where l(y) and r(y) denote the left and right side representations of a fuzzy number, respectively.

### 3.3 Integrating an AHP method with fuzzy set theory

AHP is widely used in MCDA to obtain the required weights for different criteria (Saaty, 1977, Wu, 1998, Ohta et al., 2007). It has been successfully employed in GIS-based MCDA since the early 1990s (Carver, 1991, Malczewski, 1999a, Malczewski, 1999b, Malczewski, 2004, Makropoulos et al., 2003, Marinoni, 2004, Marinoni et al., 2009). An AHP calculates the required weights associated with the relevant criterion map layers with the help of a preference matrix in which all of the identified relevant criteria are compared with each other on the basis of preference factors (Feizizadeh and Blaschke, 2013a). The weights can then be aggregated with criterion values to arrive at a single scalar value for each decision variant (e.g. each location) representing the relative strength of the given variant. The purpose of AHP is to take into account expert knowledge, and since a conventional AHP cannot properly reflect the human choice making based on quantitative articulation of preferences, a fuzzy extension of AHP (called FAHP) was developed to solve the fuzzy hierarchical problems. In the FAHP procedure, the pairwise comparisons in the judgment matrix are fuzzy numbers that are modified by the analyst (Kahraman et al., 2003). Within this study we employed the FAHP approach to fuzzify hierarchical analysis by allowing fuzzy numbers for the pairwise comparisons, in order to determine fuzzy weights. The following steps were taken after Chen et al. (2011) to determine evaluation criteria weights using an FAHP:

**Step I**: Pairwise comparison matrices were established using all the elements/criteria in the dimensions of the hierarchy system. Linguistic terms were assigned to the pairwise comparisons as follows, asking in each case, which of the two elements/criteria were more important:
  (4) $$\tilde{A}=\left[\begin{array}{llll} \tilde{1} & {\tilde{a}}_{12} & \cdots & {\tilde{a}}_{1n}\\ {\tilde{a}}_{21} & \tilde{1} & \cdots & {\tilde{a}}_{1n}\\ \vdots & \vdots & \ddots & \vdots \\ {\tilde{a}}_{n1} & {\tilde{a}}_{n2} & \cdots & \tilde{1} \end{array}\right]=\left[\begin{array}{llll} \tilde{1} & {\tilde{a}}_{12} & \cdots & {\tilde{a}}_{1n}\\ 1/{\tilde{a}}_{21} & \tilde{1} & \cdots & {\tilde{a}}_{1n}\\ \vdots & \vdots & \ddots & \vdots \\ 1/{\tilde{a}}_{n1} & 1/{\tilde{a}}_{n2} & \cdots & \tilde{1} \end{array}\right]$$
  where ${\tilde{a}}_{ij}$ measure denotes a pair of criteria *i* and *j*, let $\tilde{1}$ be (1,1,1), when *i* equal *j* (i.e. *i=j*); if $\tilde{1},\tilde{2},\;\tilde{3},\;\tilde{4},\;\tilde{5},\;\tilde{6},\tilde{7},\tilde{8},\tilde{9}\;$ measure that criterion *i* is relatively important in comparison with creation *j* and whereas ${\tilde{1}}_{-1},{\tilde{2}}_{-1},{\tilde{3}}_{-1},{\tilde{4}}_{-1},{\tilde{5}}_{-1},{\tilde{6}}_{-1},{\tilde{7}}_{-1},{\tilde{8}}_{-1},{\tilde{9}}_{-1}\;$ measure that criterion *j* is relatively more important (Hong et al., 2005, Chen et al., 2011).
**Step II**: The geometric mean technique by Buckley was used to define the fuzzy geometric mean and fuzzy weighting of each criterion (Buckley, 1985, Chen et al., 2011) as follows:
  (5) $${\tilde{r}}_{i}={\left(\;{\tilde{a}}_{i1}⊗{\tilde{a}}_{i2}⊗\cdots ⊗{\tilde{a}}_{in}\right)}^{\left(1/n\right)},\mathrm{and}\;\mathrm{then}\;{\tilde{w}}_{i}={\tilde{r}}_{i}⊗{\left({\tilde{r}}_{1}⊗\cdots ⊗{\tilde{r}}_{n}\right)}^{-1}$$
  where ${\tilde{a}}_{in}$ is the fuzzy comparison value for the pair criterion *i* and criterion *n,* ${\tilde{r}}_{i}$ is the geometric mean of the fuzzy comparison values for criterion *i* compared to each of the other criteria, and ${\tilde{w}}_{i}$ is the fuzzy weighting of the *i*th criterion, which can also be represented by a TFN, ${\tilde{w}}_{i}=(Iw_{i},mw_{i},uw_{i}),\;wh\mathrm{ere}\;Iw_{i},\;mw_{i}\;\mathrm{and}\;uw_{i}$ stand for the lower, middle and upper values, respectively, of the fuzzy weighting of the *i*th criterion, (Chen et al., 2011). In the context of FAHPs based on triangular fuzzy numbers, several approaches have been proposed (Erensal et al., 2006); for this study we employed a fuzzy extent analysis for FAHP, as detailed below.

### 3.4 Extent analysis method based on a FAHP

Chang (1996) developed an approach using triangular fuzzy numbers for the pairwise comparison scale of FAHP, and using an extent analysis method to obtain the synthetic extent values of the pairwise comparisons. Fuzzy numbers are represented by membership functions used to handle imprecise information, such as ‘close to 5′ or ‘very important’. There are various types of fuzzy numbers, any one of which may be more suitable than others for analyzing a given ambiguous structure (Sen and Cınar, 2010). In our study the extent analysis method was applied to FAHP to a landslide susceptibility problem. When the expert judgments are expressed as triangular fuzzy numbers, the triangular fuzzy comparison matrix is:

(6) $$\tilde{A}-{\left({\tilde{a}}_{ij}\right)}_{n\times n}\left[\begin{array}{llll} \left(1,1,1\right) & \left(l_{12},\;m_{12},u_{12}\right) & \cdots & (l_{1n},\;m_{1n},u_{1n})\;\\ \left(l_{21},\;m_{21},u_{21}\right) & \left(1,1,1\right) & \cdots & (l_{2n},\;m_{2n},u_{2n})\;\\ \vdots & \vdots & \ddots & \vdots \\ \left(l_{n1},\;m_{n1},u_{n1}\right) & \left(l_{n2},\;m_{n2},u_{n2}\right) & \cdots & \left(1,1,1\right) \end{array}\right]$$

where ${\tilde{a}}_{ij}=(l_{ij},m_{ij},u_{ij},)\;\mathrm{and}\;{\tilde{a}}_{ij}^{-1}=(1/u_{ij},\;1/m_{ij},\;1/l_{ij})$

(7) forI,j,−1,…,nandi≠j.

The steps of Chang’s fuzzy extent analysis can be summarized as follows (Vahidnia et al., 2009):

First, sum each row of the fuzzy comparison matrix $\tilde{A}$. Then normalize the sums of each of the rows (obtaining their fuzzy synthetic extent) using the fuzzy arithmetic operation:

(8) $${\tilde{S}}_{i}=\sum_{j}^{n}{\tilde{a}}_{ij}⊗{\left[\sum_{k-1}^{n}\sum_{j-1}^{n}{\tilde{a}}_{kj}\;\right]}^{-1}=\left(\frac{\sum_{j-1}^{n}l_{ij}}{\sum_{k=1}^{n}\sum_{j=1}^{n}u_{kj}},\frac{\sum_{j-1}^{n}m_{ij}}{\sum_{k=1}^{n}\sum_{j=1}^{n}m_{kj}},\frac{\sum_{j-1}^{n}u_{ij}}{\sum_{k=1}^{n}\sum_{j=1}^{n}l_{kj}}\right)\;,\;i-1,\ldots ,n.\;$$

where ⊗ denotes extended multiplication of two fuzzy triangular numbers. These fuzzy triangular numbers are the relative weightings for each alternative under a given criterion. They also represent the weighting ascribed to each criterion with respect to the overall objective. A weighted summation is then used to obtain the overall performance of each alternative (Vahidnia et al., 2009).

Second, compute the degree of possibility for ${\tilde{S}}_{i}\geq {\tilde{S}}_{j}$ using the following equation:

(9) $$V({\tilde{S}}_{i}\geq \;{\tilde{S}}_{j})\;-\;{\;}_{y\geq x}^{\sup}[\;\min({\tilde{S}}_{i}(x),{\tilde{S}}_{i}(y)]$$

This formula can also be expressed as:

(10) $$V({\tilde{S}}_{i}\geq \;{\tilde{S}}_{j}\;)-\{\begin{array}{ll} 1 & m_{i}\geq \;m_{j\;}\\ \frac{u_{i-li}}{\left(u_{i-m_{i}})+(m_{i-l_{i}}\right)} & l_{j}\leq \;u_{i}i,\;j-1,\ldots ,n;j\neq i\\ 0 & \mathrm{otherwise} \end{array}$$

where

(11) $${\tilde{S}}_{i}\geq {\tilde{S}}_{j}-(l_{i\;},m_{i\;},u_{i})\;\mathrm{and}\;{\tilde{S}}_{j}-(l_{j\;},m_{j\;},u_{j})$$

**Fig. 4:**
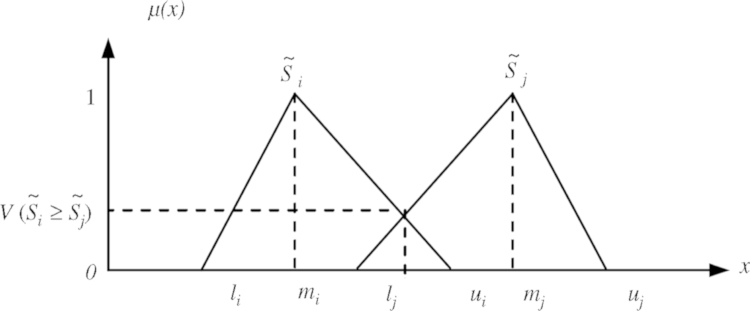
The degree of possibility $V({\tilde{S}}_{i}\geq {\tilde{S}}_{j})$ (Vahidnia et al., 2009).

Fig. 4 illustrates this degree of possibility for two fuzzy numbers.

Finally, estimate the priority vector of the

(12) $$W{\left(w_{1},\ldots ,w_{n}\right)}^{T}$$

fuzzy comparison matrix $\tilde{A}$ as follows:

(13) $$w_{i}-\frac{V({\tilde{S}}_{i}\geq {\tilde{S}}_{j}|j-1,\ldots ,n,j\neq i)}{\sum_{k-1}^{n}V({\tilde{S}}_{k}\geq {\tilde{S}}_{j}|j-1,\ldots ,n;j\neq k),}i-1,\ldots ,n$$

**Fig. 5:**
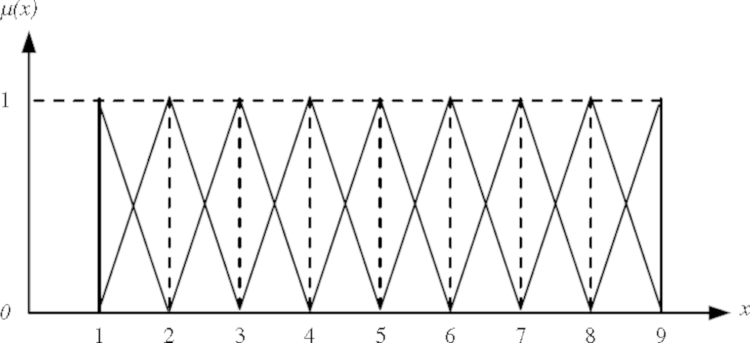
TFNs corresponding to linguistic variables representing levels of preference (Vahidnia et al., 2009).

In order to perform a pairwise comparison between fuzzy parameters, we defined linguistic variables for several levels of preference (see Table 1). The fuzzy triangular numbers were used to represent these preferences, which are depicted in Fig. 5.

**Table 1:** Triangular fuzzy number of linguistic variables used in this study (Saaty and Vargas, 2008, Vahidnia et al., 2009).

Linguistic variables	Triangular fuzzy numbers	Reciprocal triangular fuzzy numbers
Extremely strong	(9,9,9)	(1/9, 1/9, 1/9)
Very strong	(6,7,8)	(1/8, 1/7, 1/6)
Strong	(4,5,6)	(1/6, 1/5, 1/4)
Moderately strong	(2,3,4)	(1/4, 1/3, 1/2)
Equally strong	(1,1,1)	(1,1,1)
Intermediate	(7,8,9), (5,6,7), (3,4,5), (1,2,3)	(1/9, 1/8,1/7), (1/7, 1/6,1/5), (1/5, 1/4,1/3), (1/3, 1/2,1)

When a pair *(x,y)* exists such that x≥y and $\mu M_{1}(x)=\mu M_{2}(y)$, we then have $V(M_{1}\geq M_{2}){=}^{\;}1.$

Since *M*_1_and *M*_2_ are convex fuzzy numbers we have that:

(14) $$\{\begin{array}{ll} V(M_{1}\geq M_{2})=1 & \;\overset{if}{\to }M_{1}\geq M_{2}\\ V(M_{1}\geq M_{2})=hgt\;(M_{1}\cap M_{2})=\mu M_{1}(d)\; & \;otherwise \end{array}$$

where *d* is the ordinate of the highest intersection point *D* between $\mu M_{1}$ and $\mu M_{2}$. The ordinate of *D* is defined as follows:

(15) $$V(M_{1}\geq M_{2})=hgt\;(M_{1}\cap M_{2})=\;\mu M_{1}(d)\;\frac{m_{1}-m_{3}}{\left({m\prime }_{2}-{m\prime }_{3})+(m_{2}-m_{3}\right)}$$

To compare $M_{1}\;\mathrm{and}\;M_{2},$ the value of $V(M_{1}\geq \;M_{2})$ first needs to be calculated. The degree of possibility for a convex fuzzy number to be greater than *k* convex fuzzy numbers $M_{i}(i=1,2,\ldots ,n_{\;})$ can be defined by:

(16) $$V(M\geq M_{1,}M_{2,}\ldots ,M_{k})=\;\min(V(M\geq M_{i}))\;i=1,2,\ldots ,k$$

assuming that

(17) $$W\prime (A_{i})\min\;\{V(S_{i}\geq S_{k})\}\;k=1,2,\ldots ,n;\;k\neq i$$

The weighting vector can then be computed by:

(18) $$W\prime (A_{i})={\left[W\prime (A_{1}),\;W\prime (A_{2}),\;\ldots ,\;W\prime (A_{n})\;\right]}^{T}$$

where $A_{i}(i=1,2,\ldots ,n)\;\mathrm{are}\;n\;\mathrm{elements}.$ Following normalization, the normalized weight vectors are:

(19) $$W(A_{i})={\left[W(A_{1}),W(A_{2}),\;\ldots ,\;W(A_{n})\right]}^{T}$$

where *W* is considered to be a nonfuzzy number.

### 3.5 Fuzzy synthetic decision

In FAHP the weighting ascribed to each criterion and the fuzzy performance values must be integrated by the calculation of fuzzy numbers so as to be located at the fuzzy performance value (effect-value) of the integral evaluation. The criteria weight vector $\tilde{w}={\left({\tilde{w}}_{1},\ldots ,{\tilde{w}}_{i},\ldots ,{\tilde{w}}_{n}\right)}^{t}$can be obtained using each of the criterion weightings (${\tilde{w}}_{i}$) derived by the FAHP, while the fuzzy performance/evaluation matrix $\tilde{E}$ for each of the alternatives can be obtained from the fuzzy performance value of each alternative under *n* criteria, that is, $\tilde{E}={\left(e_{ki}\right)}_{m\times n}.$ A final fuzzy synthetic decision can be derived from the criteria weighting vector $\tilde{w}$ and the fuzzy performance matrix $\tilde{E}$, the result being in the form of a fuzzy synthetic decision vector $\tilde{e}\;=(e_{1},\ldots ,\;e_{k},\ldots ,e_{m})\prime$ (Chen et al., 2011), that is:

(20) $$\tilde{e}=\;\tilde{E}⊗\tilde{w}=\tilde{w}\prime ⊗\tilde{E}\prime$$

in which the sign ⊗ indicates the calculation of the fuzzy numbers, including fuzzy addition and fuzzy multiplication. Since fuzzy multiplication is rather complex, it is usually denoted by the approximate result of the fuzzy multiplication. The approximate fuzzy number ${\tilde{S}}_{i}$ from the fuzzy synthetic decision of each alternative can be shown as

(21) $$\;e_{k}={\left(le_{k},\;me_{k},ue_{k}\right)}_{\;}$$

where $ls_{k}$, $\;ms_{k}$ and $us_{k}$ are the lower, middle and upper synthetic performance values, respectively, of alternative *k* (Chen et al., 2011), that is:

(22) $$ls_{k}=\sum_{i=1}^{n}le_{ki}\times lw_{i},\;me_{k}=\sum_{i=1}^{n}me_{ki}\times mw_{i},\;ue_{k}=\sum_{i=1}^{n}ue_{ki}\times uw_{i}$$

### 3.6 Ranking the fuzzy numbers

Fuzzy number is the results of fuzzy synthetic decision attained by the various alternatives. In order to compare the respective fuzzy number for determining the most effective alternative plans a defuzzification method is applied (Opricovic and zengG, 2003, Chen et al., 2011). Methods used for defuzzification of such fuzzy rankings generally include mean of maximal (MOM), center of area (COA), and a-cut methods. The COA method offers a simple and practical way to determine the BNP, with no need to include the preferences of any evaluators. The BNP value of the fuzzy number ${\tilde{e}}_{k}$, which is equal to $(le_{k},\;me_{k},ue_{k}),$ can be found using the following equation:

(23) $$\;BNP=le_{k}+\frac{\left(ue_{k}-le_{k})+(me_{k}-le_{k}\right)}{3},\;\forall k.$$

Based on the achieved BNP value for each alternative, a respective ranking of the best plan for alternatives can then be applied (Chen et al., 2011).

## 4 Application of an FAHP combined with fuzzy standardization for LSM

**Fig. 6:**
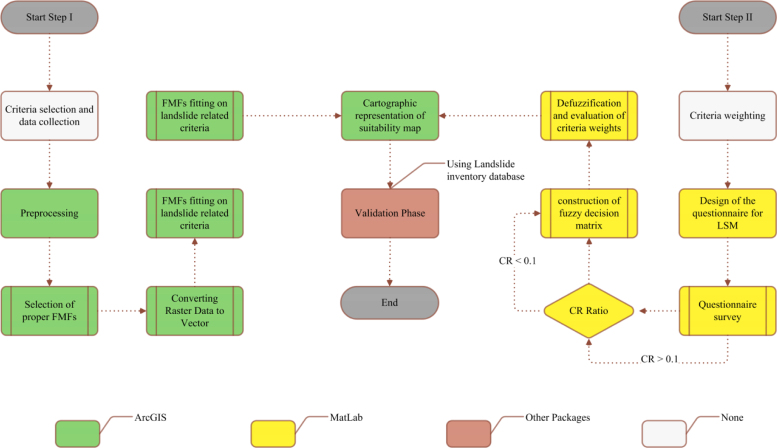
Schematic representation of proposed LSM.

The integration of fuzzy sets with GIS-MCDA has been demonstrated to be an effective methodology for susceptibility assessment and hazard mapping (Mason and Rosenbaum, 2002). Also, applying a FMF in standardization process for LSM allows to establishing the susceptibility degree of landslides occurrences for any individual pixel within each criteria. Pixels can be attributed numeric values ranging from 0 (not susceptible) to 1 (very susceptible) (Pourghasemi et al., 2012). In our study we implemented a two-stage FAHP for LSM as follows (see Fig. 6).

### 4.1 Stage I: Using FAHP to determine criteria weights

Criteria weights were first assigned to the attribute maps (Meng et al.,, Feizizadeh and Blaschke, 2013b, Feizizadeh et al., 2013b). The derivation of weights is a central step in eliciting the decision Maker’s preferences (Malczewski, 2000). A pairwise comparison matrix was then established using the prior knowledge of goodness-of-fit in order to assign weights before producing a landslide susceptibility map. The standardized predictor variable values were aggregated with weights derived from FAHP in order to evaluate the sensitivity of the landslide susceptibility map to different predictor variables. Table 2 shows the FAHP-based pairwise comparison matrix calculated for nine criteria. In order to obtain final criteria weights from the FAHP, the synthetic values (see Section 3.5) were first calculated as an FAHP pairwise matrix (et al., 2003):

(24) $${\left[\sum_{i=1}^{n}\sum_{j=1}^{m}M_{g_{i}}^{j}\;\right]}^{-1}={\left(74.18\;105.98\;163.15\right)}^{-1}=(0.006\;0.009\;0.013)$$

*S*_*slope*_=(15.4 24.8 34.5)×(0.006 0.009 0.013)=(0.09 0.23 0.47), *S*_*aspect*_=(4.3 6.7 9.1)×(0.006 0.009 0.013)=(0.02 0.06 0.12), *S*_*distance to streams*_=(8.3 12.7 23)×(0.006 0.009 0.013)=(0.05 0.1211 0.31), *S*_*drainage density*_=(6.2 9.2 15.3)×(0.006 0.009 0.013)=(0.03 0.08 0.20), *S*_*distance to faults*_=(4.8 7.5 13)×(0.006 0.009 0.013)=(0.03 0.07 0.17), S_*precipitation*_=(3.7 5.1 8.1)×(0.006 0.009 0.013)=(0.02 0.05 0.11), *S*_*distance to roads*_=(5.75 13.5 19.1)×(0.006 0.009 0.013)=(0.03 0.12 0.26), S_*lithology*_=(13.1 19.4 29.2)×(0.006 0.009 0.013)=(0.08 0.18 0.4), *S*_*land use/cover*_=(5 6.7 11.7)×(0.006 0.009 0.013)=(0.03 0.06 0.16).

**Table 2:** FAHP evaluation matrix.

	1	2	3	4	5	6	7	8	9
Slope (1)									
*M*_1_	1	2.5	2	2	1.3	2	1.6	0.9	2
*M*_2_	1	3.5	3.5	2.75	2.5	4	2.85	1.75	3
*M*_3_	1	4.5	4	3.65	3.7	5	5	2.65	5
Aspect (2)									
*M*_1_	0.22	1	0.25	0.5	0.5	0.8	0.28	0.25	0.5
*M*_2_	0.28	1	0.4	0.66	1.5	1.25	0.4	0.33	0.9
*M*_3_	0.4	1	0.5	1	2	1.6	1	0.5	1.1
Distance to streams (3)									
*M*_1_	0.25	2	1	0.54	0.5	1.5	0.67	0.4	1
*M*_2_	0.28	2.5	1	1	1.5	2.5	1.33	0.67	2
*M*_3_	0.5	4	1	2	2	5	4	1.5	3
Drainage density (4)									
*M*_1_	0.27	1	0.5	1	0.75	1	0.67	0.28	0.75
*M*_2_	0.36	1.5	1	1	1	1.54	1	0.35	1.5
*M*_3_	0.5	2	1.85	1	3	2.5	2	0.45	2
Distance to fault (5)									
*M*_1_	0.27	0.5	0.5	0.33	1	1	0.25	0.25	0.7
*M*_2_	0.4	0.66	0.66	1	1	2	0.33	0.5	1
*M*_3_	0.75	2	1	1.33	1	3	1	0.75	2.2
Precipitation (6)									
*M*_1_	0.2	0.62	0.2	0.4	0.33	1	0.25	0.2	0.5
*M*_2_	0.25	0.8	0.4	0.65	0.5	1	0.4	0.33	0.75
*M*_3_	0.5	1.25	0.66	1	1	1	1	0.67	1
Distance to road (7)									
*M*_1_	0.2	1	0.25	0.5	1	1	1	0.3	0.5
*M*_2_	0.35	2.5	0.75	1	0.3	2.5	1	0.4	2
*M*_3_	0.6	3.5	1.5	1.5	0.4	4	1	0.5	2.5
Lithology (8)									
*M*_1_	0.37	2	0.6	2.2	1.33	1.5	2	1	2
*M*_2_	0.57	3	1.5	2.8	2	3	2.5	1	3
*M*_3_	1.1	4	3.3	3.5	4	5	3.33	1	4
Land use/cover (9)									
*M*_1_	0. 2	0.9	0.33	0.5	0.45	1	0.4	0.25	1
*M*_2_	0.33	1.1	0.5	0.66	1	1.33	0.5	0.33	1
*M*_3_	0.5	2	1	1.3	1.43	2	2	0.5	1

In making the calculations, the fuzzy values were normalized as in Eq. (19). The results of this stage are shown in Table 3. The weight vector was then calculated from Table 3 as follows:

(25) $$W\prime (x_{i})={\left\{1\;0.33\;0.613\;0.478\;0.434\;0.294\;0.612\;0.708\;0.39\right\}}^{T}$$

(26) $$W\prime (x_{i})={\left\{0.177\;0.07\;0.13\;0.101\;0.092\;0.062\;0.131\;0.15\;0.083\right\}}^{T}$$

**Table 3:** The ordinate of the highest intersection point and the degree possibility for TFNs.

*i*=Slope	*i*=Aspect	*i*=Distance to stream
*V*($S_{i}\geq S_{Aspect}$)=1	*V*($S_{i}\geq S_{Slope}$)=0.330	*V*($S_{i}\geq S_{Slope}$)=0.613
*V*($S_{i}\geq S_{Stream}$)=1	*V*($S_{i}\geq S_{Stream}$)=0.386	*V*($S_{i}\geq S_{Aspect}$)=1
*V*($S_{i}\geq S_{Drainage}$)=1	*V*($S_{i}\geq S_{Drainage}$)=0.538	*V*($S_{i}\geq S_{Drainage}$)=1
*V*($S_{i}\geq S_{Fault}$)=1	*V*($S_{i}\geq S_{Fault}$)=0.587	*V*($S_{i}\geq S_{Fault}$)=1
*V*($S_{i}\geq S_{\mathrm{Pr}ecipitation}$)=1	*V*($S_{i}\geq S_{\mathrm{Pr}ecipitation}$)=1	*V*($S_{i}\geq S_{\mathrm{Pr}ecipitation}$)=1
*V*($S_{i}\geq S_{Road}$)=1	*V*($S_{i}\geq S_{Road}$)=0.506	*V*($S_{i}\geq S_{Road}$)=0.806
*V*($S_{i}\geq S_{Litho\log y}$)=1	*V*($S_{i}\geq S_{Road}$)=0.354	*V*($S_{i}\geq S_{Litho\log y}$)=0.643
*V*($S_{i}\geq S_{Landuse}$)=1	*V*($S_{i}\geq S_{Landuse}$)=0.624	*V*($S_{i}\geq S_{Landuse}$)=1

$\min\;\{V(S_{i}\geq S_{k})\}$=1	$\min\;\{V(S_{i}\geq S_{k})\}$=0.330	$\min\;\{V(S_{i}\geq S_{k})\}$=0.613

*i*=Drainage density	*i*=Distance to fault	*i*=Precipitation

*V*($S_{i}\geq S_{Slope}$)=0.478	*V*($S_{i}\geq S_{Slope}$)=0.434	*V*($S_{i}\geq S_{Slope}$)=0.294
*V*($S_{i}\geq S_{Aspect}$)=1	*V*($S_{i}\geq S_{Aspect}$)=1	*V*($S_{i}\geq S_{Aspect}$)=0.714
*V*($S_{i}\geq S_{Stream}$)=0.750	*V*($S_{i}\geq S_{Stream}$)=0.461	*V*($S_{i}\geq S_{Stream}$)=0.294
*V*($S_{i}\geq S_{Fault}$)=1	*V*($S_{i}\geq S_{Drainage}$)=0.652	*V*($S_{i}\geq S_{Drainage}$)=0.478
*V*($S_{i}\geq S_{\mathrm{Pr}ecipitation}$)=1	*V*($S_{i}\geq S_{\mathrm{Pr}ecipitation}$)=1	*V*($S_{i}\geq S_{\mathrm{Pr}ecipitation}$)=0.520
*V*($S_{i}\geq S_{Road}$)=0.672	*V*($S_{i}\geq S_{Road}$)=0.621	*V*($S_{i}\geq S_{Road}$)=0.449
*V*($S_{i}\geq S_{Litho\log y}$)=0.506	*V*($S_{i}\geq S_{Litho\log y}$)=0.461	*V*($S_{i}\geq S_{Litho\log y}$)=0.315
*V*($S_{i}\geq S_{Landuse}$)=1	*V*($S_{i}\geq S_{Landuse}$)=1	*V*($S_{i}\geq S_{Landuse}$)=0.552

$\min\;\{V(S_{i}\geq S_{k})\}$=0.478	$\min\;\{V(S_{i}\geq S_{k})\}$=0.434	$\min\;\{V(S_{i}\geq S_{k})\}$=0.294

*i*=Distance to road	*i*=Lithology	*i*=Land use/cover

*V*($S_{i}\geq S_{Slope}$)=0.612	*V*($S_{i}\geq S_{Slope}$)=0.708	*V*($S_{i}\geq S_{Slope}$)=0.390
*V*($S_{i}\geq S_{Aspect}$)=1	*V*($S_{i}\geq S_{Aspect}$)=1	*V*($S_{i}\geq S_{Aspect}$)=1
*V*($S_{i}\geq S_{Stream}$)=1	*V*($S_{i}\geq S_{River}$)=1	*V*($S_{i}\geq S_{Stream}$)=0.448
*V*($S_{i}\geq S_{Drainage}$)=1	*V*($S_{i}\geq S_{Drainage}$)=1	*V*($S_{i}\geq S_{Drainage}$)=0.595
*V*($S_{i}\geq S_{Fault}$)=1	*V*($S_{i}\geq S_{Fault}$)=1	*V*($S_{i}\geq S_{Fault}$)=0.6399
*V*($S_{i}\geq S_{\mathrm{Pr}ecipitation}$)=1	*V*($S_{i}\geq S_{\mathrm{Pr}ecipitation}$)=1	*V*($S_{i}\geq S_{\mathrm{Pr}ecipitation}$)=1
*V*($S_{i}\geq S_{Litho\log y}$)=0.647	*V*($S_{i}\geq S_{Road}$)=1	*V*($S_{i}\geq S_{Road}$)=0.565
*V*($S_{i}\geq S_{Landuse}$)=1		*V*($S_{i}\geq S_{Litho\log y}$)=0.415
$\min\;\{V(S_{i}\geq S_{k})$=0.612	$\min\;\{V(S_{i}\geq S_{k})\}$=0.708	$\min\;\{V(S_{i}\geq S_{k})\}$=0.390

This defuzzification process resulted in crisp weights, which were then used for LSM criteria integration (see Table 4). From these results slope and lithology were identified as the two most important criteria for LSM.

**Table 4:** The calculated weight vector from FAHP and TFNs.

Criteria	Weight
Slope	0.177
Aspect	0.07
Distance to stream	0.13
Drainage density	0.101
Distance to fault	0. 092
Precipitation	0.062
Distance to roads	0.131
Lithology	0.15
Land use/cover	0.083

### 4.2 Stage II: Application of FMFs

**Fig. 7:**
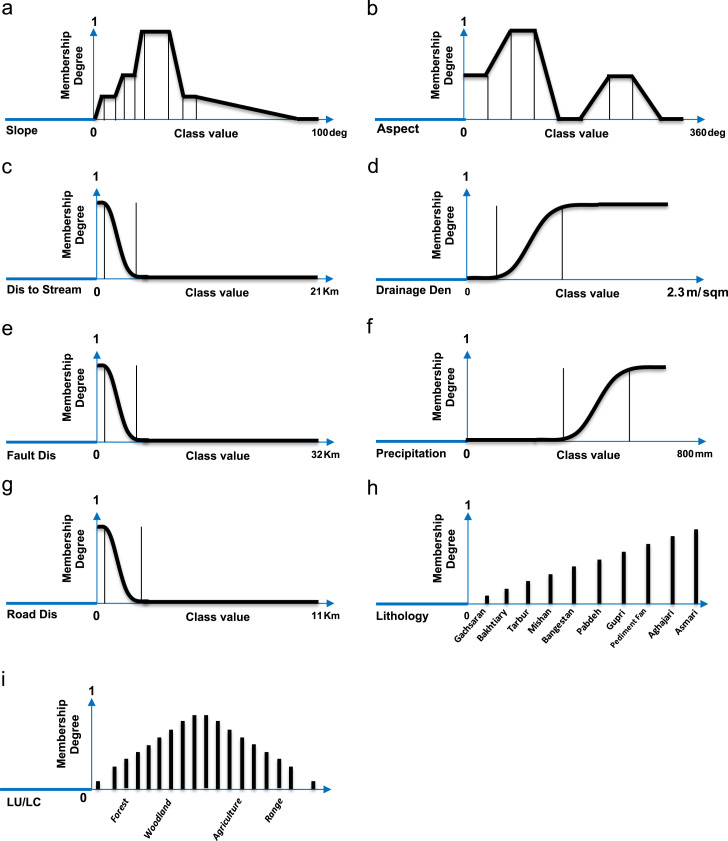
FAHP-based membership functions including: (Type I) user defined FMFs for (a) slope and (b) aspect, (Type II) Sigmoidal FMFs for (c) distance to streams, (d) drainage density, (e) distance to faults, (f) precipitation, (g) distance to roads, and (Type III) Crisp MFs for (h) lithology and (i) land use/cover.

In the second stage all criteria were standardized using a fuzzy set. In order to standardize landslide related criteria in GIS framework, base on the defined fuzzy and crisp membership functions (see Fig. 7), nine raster datasets are first constructed for each landslide related criteria based on subsequent slope, aspect, distance, density, polygon to raster and kriging interpolation functions (see Fig. 2). Afterwards, cell values of each raster datasets associated with each landslide related criteria are converted to fuzzy scores using raster calculator in ArcGIS environment. In essence, these fuzzy scores are fuzzy membership values attaches to each cell (which range from the least susceptible 0 to the most susceptible 1).

**Fig. 8:**
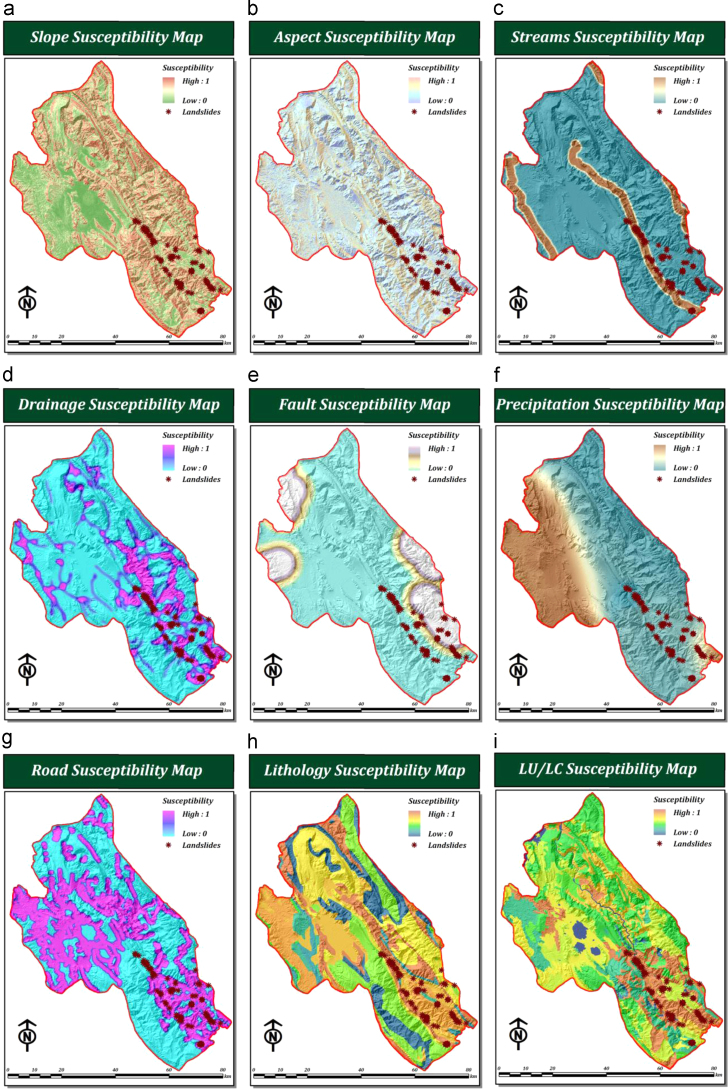
Spatial distribution of landslide susceptibility for each criterion, based on fuzzy membership functions (i.e. fuzzy or crisp) of each parameter: (a) slope, (b) aspect, (c) distance to streams, (d) drainage density, (e) distance to faults, (f) precipitation, (g) distance to roads, (h) lithology, and (i) land use/cover.

There is no optimal method for choosing the most appropriate FMFs and their respective parameters and they are generally selected according to the preferences of the decision makers or analyst experience. In this process, sigmoidal membership functions (i.e., monotonically decreasing and monotonically increasing), user-defined linear membership functions, two crisp membership functions, are specified for selected landslide criteria (see Fig. 7). The sigmoidal membership function is probably the most commonly used function in fuzzy set theory (Zadeh, 1965, Eastman, 2004, Liu et al., 2004). although user-defined linear FMFs or crisp membership functions are also sometimes used. Here, regarding the inherent characteristics of lithology and land use/land cover criteria two different crisp membership function (i.e. two look up table) were implemented for further standardization of those mentioned criteria (see Fig. 7). To this end, less susceptibility value is assigned to the less susceptible formation or land used/ land cover class, and vice versa. All membership functions obtained from LSM criteria outputs are applied to each parameter, which are then classified into groups on the basis of their landslide susceptibilities (see Fig. 8).

## 5 Results and validation

**Fig. 9:**
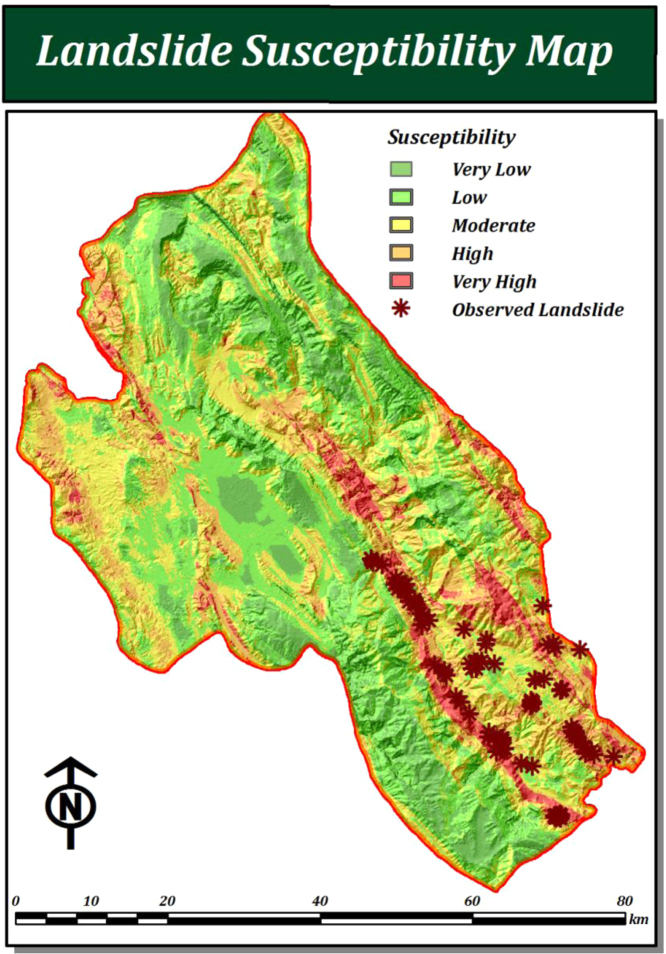
Final landslide susceptibility map.

The final landslide susceptibility map was produced using the results from the two stages described in Section 4 above, in the following way:

(27) $$LSM_{AHP}=(slope\;\deg ree\times W_{AHP})+(aspect\times W_{AHP})+(dis\;\tan\;ce\;to\;stream+W_{AHP})+(drainage\;density+W_{AHP})+(distance\;to\;faults+W_{AHP})+(precipitation+W_{AHP})+(distance\;to\;roads+W_{AHP})+(Lithology+W_{AHP})+(Land\;us/\mathrm{cov}er+W_{AHP})$$

where *W*_AHP_ is the respective weight for the each of the LSM criteria. The resulting landslide susceptibility map was then divided into five susceptibility categories (very low, low, moderate, high, and very high) using the natural breaks method to determine the class intervals (Feizizadeh and Blaschke., 2013a) (see Fig. 9).

Considering the fact that modelling is one of the main tools for the assessment of natural hazards (Nefeslioglu et al., 2013), validation is a fundamental step in the development of a susceptibility map and is important for determining its predictive ability. Accordingly, the final landslide susceptibility map was validated using known landslide locations (Yilmaz, 2010). The predictive capability of landslide susceptibility maps is usually estimated using independent information that was not utilized in the LSM process. The accuracy of the landslide susceptibility map was therefore evaluated through the relative operating characteristics (ROCs) (Fawcett, 2006, Nandi and Shakoor, 2009), by analyzing known landslides that have been observed within each of the various categories of the landslide susceptibility map.

**Fig. 10:**
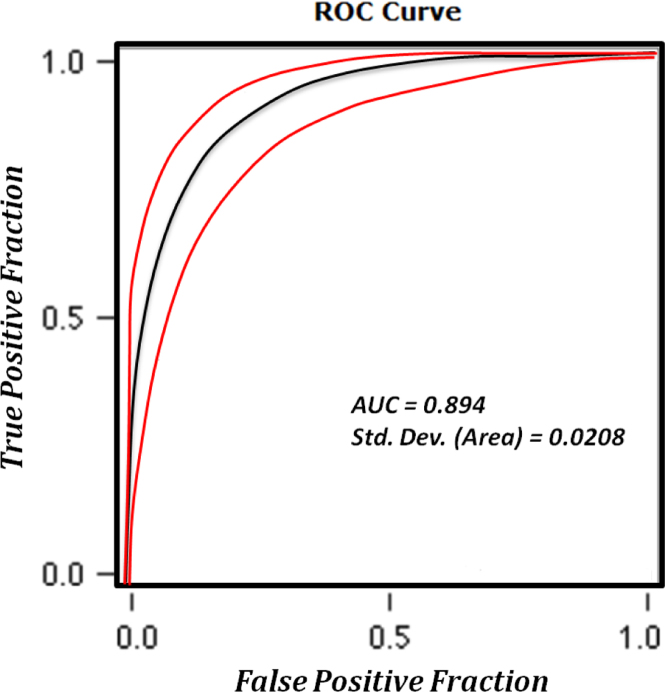
ROC curve for the obtained landslide susceptibility map.

**Fig. 11:**
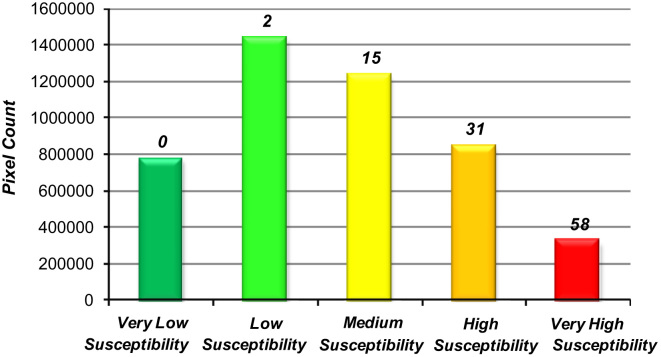
Validation of landslide susceptibility map using known landslides in the study area.

In the ROC method, the area under the curve (AUC) values (which range from 0.5 to 1.0) are base of accuracy assessment for the model. The AUC leads to determine the quality of the probabilistic model by describing its ability to reliably predict the occurrence or non-occurrence of landslide event. In this approach, the ideal model shows an AUC close to 1.0, while a value close to 0.5 indicates inaccuracy in the model (Fawcett, 2006, Nandi and Shakoor, 2009, Feizizadeh et al., 2013c). In order to apply the ROC method, a representative dataset was prepared based on the landslide inventory database. Accordingly, to compute the AUC, 106 known landslide events were used and 108 non-landslide locations were selected at random. The AUC of the ROC curve was calculated to be 0.894 with a standard error of 0.02 (see Fig. 10). The resulting landslide susceptibility map was also verified using the landslide inventory map, by overlaying the 106 known landslides on the landslide susceptibility map (see Fig. 11). Approximately 84% of known landslides fell in the ‘very high susceptibility’ and ‘high susceptibility’ zones, which together cover 25% of the total study area. Almost 14.15% of known landslides fell into the ‘moderate susceptibility’ category, with only 1.89% of landslides falling in the ‘low susceptibility category’. No landslide events were recorded from within the ‘very low susceptibility’ category.

## 6 Discussion

Our research aims to integrate fuzzy set theory with AHP-MCDA for LSM. We introduced an approach that integrates fuzzy set theory and information theory algorithms (i.e. extended), which could be a useful geospatial tool for integrating multiple features/attributes that affect the LSM process. This is an integrated strategic LSM framework with emphasis on structuring the decision making process problem. Within this approach a FAHP was employed to determine the criteria weightings from subjective judgments of decision-making domain experts. This FAHP approach includes careful selection and standardization of landslide related criteria and weighting procedures using objective methods which determine the criteria weights by solving mathematical models without any consideration of the decision maker’s preferences (as is conventional in subjective methods). The results confirm that the integration of fuzzy set theory with AHP can result in high-reliability landslide susceptibility maps. This Fuzzy-AHP integration is promising for GIS-MCDA as it tackles two major limitations of the traditional AHP. Firstly, AHP is usually applied in a single process, relying on expert knowledge for assessing the criteria weights while allowing a certain degree of subjectivity in the pairwise comparison matrix. Secondly, the incompatibility of the technique with rational choice theory has been ascribed to a limited scale of judgment, lack of transitivity, and the rank reversal phenomenon. Although several alternative scales have been recommended, none of them completely address the above mentioned problems with AHP. Saaty (1977) used a ratio scale which is used by nearly all applications (Duru et al., 2012). The uncertainty of information and the vagueness of human judgment make it difficult to provide exact numerical weights for evaluation criteria. Most of the pairwise comparison ratings cannot be selected precisely and experts may therefore prefer intermediate ratings rather than certain ratings. To overcome this lack of precision, FAHP makes the comparison process more flexible for eliciting experts’ preferences (Kahraman et al., 2003, Kutlu and Ekmekçioglu, 2012). In the FAHP approach, every choice has its own particular regime, which is associated with a two-dimensional priority matrix (e.g. criteria vs. criteria). On the other hand, conventional AHP uses pairwise comparisons of criteria in a top-down process and weights choice matrices by the result of a single identical priority matrix (Duru et al., 2012). Since the evaluation criteria of the best plan have the diverse connotations and meanings, there is no logical reason to treat them all as being of equal importance. Furthermore, FAHP was used to handle the qualitative criteria of LSM (e.g. land use, aspect, and lithology) which are difficult to express in crisp values, thus strengthening the approach and making it more versatile and accommodating to different ways of expressing preferences (Chen et al., 2011).

**Fig. 12:**
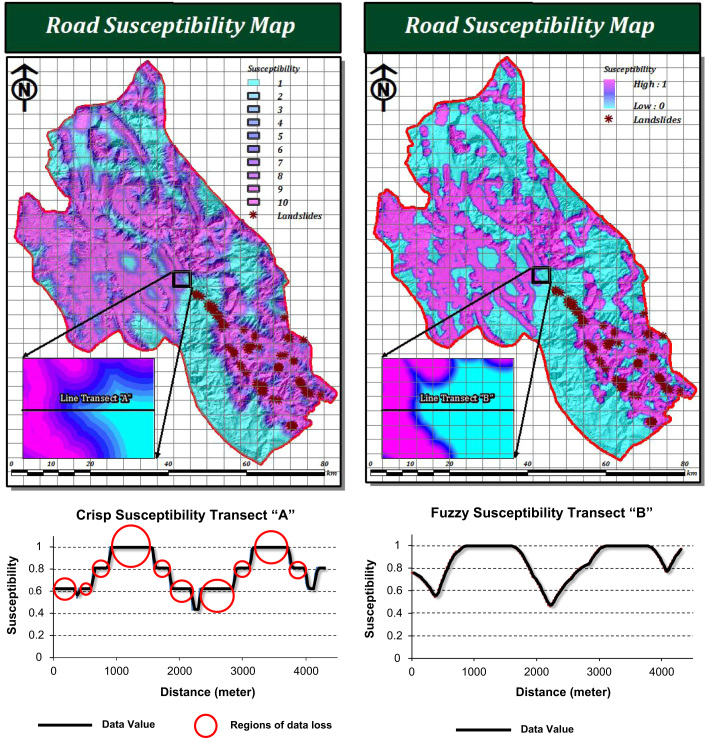
Illustration of data loss due to crisp standardization process in geographic information systems.

FAHP evaluates both priorities and data through fuzzy sets (Duru et al., 2012). In our research, the extended FAHP framework has been applied to LSM. The innovation for LSM research is that this framework uses synthetic extent values derived through pairwise comparisons. However, as Duru et al. (2012) point out, many FAHP studies ignore the matrix consistency problem, even if the judgments are inconsistent. Our results indicate that the integration of fuzzy sets with AHP in both criteria weighting and standardization leads to greater flexibility in judgment and decision making. In fact, this method addresses uncertainties in LSM by (a) using FMFs in the susceptibility model, and (b) using TFNs instead of crisp numbers when comparing the relative importance of the various LSM criteria (Chen et al., 2011). Using more computationally intensive FMFs preserve the original quality of spatial data. In this respect using variety of FMFs positively affect validity and accuracy of input spatial criteria. Missing values, or generalized inputs, can appear in otherwise precise data. Fig. 12 illustrates data loss due to using crisp standardization (i.e. reclassification) in geographic information systems.

These important functionalities are not supported by conventional AHP, which assumes that the relative importance of criteria remains identical for every decision alternative (Duru et al., 2012). In contrast, FAHP uses a range of values to express the decision maker’s uncertainty. Obviously removing uncertainty from decision making models leads to an improved accuracy of results. In this regard, Feizizadeh and Blaschke (2014) pointed out that the traditional AHP suffers from sensitivity in decision making and is prone to error of expert knowledge. They demonstrated that removing uncertainty from AHP’s weights by applying Monte Carlo simulation tends to lead to more accurate results. As AHP’s pairwise matrix represents a Boolean framework for criteria ranking, obviously its integration with fuzzy approach leads to an improved decision making approach. According to our results it can be stated the AHP is a very well suited methodology to evaluate LSM maps while integrated into GIS-MCDA. This holds true for both criteria weighting and standardization, taking uncertainties into account in the LSM process not only by using FMFs in susceptibility modeling but also by means of TFNs instead of crisp numbers for comparing the relative importance between LSM criteria. On the other hand, fuzzy logic is attractive because it is straightforward to understand and implement. It can be used with data from any measurement scale, and the weighting of evidence is controlled entirely by the expert (Feizizadeh et al., 2013b). Nevertheless, using linguistic variables makes the evaluation process more realistic. Because evaluation is not an exact process and has fuzziness in its body. Here, the use of FAHP weights makes the application more realistic and reliable (Oguzitimur, 2011).

## 7 Conclusion and future work

This study presents an integrated strategy for LSM with an emphasis on structuring the decision problem. This includes careful selection and weighting of criteria and alternative evaluations. The presented GIS-based fuzzy-MCDA framework was applied to landslide hazard, in order to understand the processes that contribute to the landslides. Our results indicate that the GIS-based fuzzy-MCDA framework offers flexibility in handling basic elements of complex decision-making problems involved in LSM. We conclude that, when compared with conventional GIS-based AHP, the FAHP framework offers greater flexibility for evaluating LSM results. There remain, however, different uncertainty aspects of LSM to be dealt with. There will always be a degree of uncertainty in any LSM as a result of the uncertainty inherent in various LSM criteria, both in the relative importance of the criteria and in the degree of landslide susceptibility indicated by each criterion. Our results show that the integration of fuzzy sets with AHP can contribute to the production of landslide susceptibility maps with a reasonably high level of reliability. To account for spatial uncertainty in FAHP approach, our future research will include the application of a spatially-explicit reliability model for spatial sensitivity and uncertainty analysis associated with AHP and FAHP. The integration of fuzzy sets with GIS-MCDA-ordered weighted averaging and uncertainty analysis of the results based on Dempster Shafer theory will also be addressed in future work. In this regard, we emphasize the importance of accuracy in landslide susceptibility maps, in order for these maps to be used as a basis for land use planning and mitigating future landslide hazards. The proposed FAHP method has the advantage of objective weight evaluation; we conclude that it can be used not only in similar areas of geo-hazards risk analysis and mapping, such as LSM, earthquake and flood risk mapping, but also in multi-hazard risk assessment for further combination of risk elements. We may emphasis that the landslide susceptibility maps with a high level of reliability are clearly important when seeking to explain the driving factors behind known landslides, as well as for supporting emergency decisions and efforts to mitigate future landslide hazards (Feizizadeh and Blaschke., 2012). The results of this study will be passed on to regional authorities in order to assist citizens, planners, and engineers to reduce the losses caused by future landslides through prevention, mitigation, and avoidance. In conjunction with our earlier research, these results will be useful in explaining the relationship between known landslides and landslide susceptibility, and can therefore be used to support decisions relating to emergency planning and mitigation in the Khuzestan province, as well as supporting the development of a landslide risk management strategy for Izeh Basin.
